# Changes of Colorectal Cancer Diagnostics and Hospitalizations during First Wave of COVID-19 Pandemic in Lithuania

**DOI:** 10.15388/Amed.2023.30.1.4

**Published:** 2023-02-23

**Authors:** Dignė Jurkevičiūtė, Sandra Mauravičiūtė, Audrius Dulskas, Inga Kildušienė, Eugenijus Stratilatovas, Sonata Jarmalaitė

**Affiliations:** Faculty of Medicine, Vilnius University, 21 M. K. Ciurlionio Str., LT-03101, Vilnius, Lithuania; Faculty of Medicine, Vilnius University, 21 M. K. Ciurlionio Str., LT-03101, Vilnius, Lithuania; Faculty of Medicine, Vilnius University, 21 M. K. Ciurlionio Str., LT-03101, Vilnius, Lithuania; National Cancer Institute, 1 Santariskiu Str., LT-08406, Vilnius, Lithuania; SMK, University of Applied Social Sciences, 137E Kalvariju Str., LT-08211, Vilnius, Lithuania; National Cancer Institute, 1 Santariskiu Str., LT-08406, Vilnius, Lithuania; National Cancer Institute, 1 Santariskiu Str., LT-08406, Vilnius, Lithuania; National Cancer Institute, 1 Santariskiu Str., LT-08406, Vilnius, Lithuania

**Keywords:** COVID-19 pandemic, colorectal cancer, colorectal cancer screening, short-term results

## Abstract

**Purpose:**

Our aim was to see the possible effect of the first COVID pandemic wave in Lithuania on colorectal cancer (CRC) preventive, diagnostic and treatment procedures.

**Methods:**

A retrospective analysis was performed using the database of the National Cancer Institute, Lithuania. We have divided patients into two groups: group 1 – patients treated during the nonpandemic period (2019 January 1 to 2019 July 31) and group 2 – the pandemic period (2020 January 1 to 2020 July 31). We analyzed numbers of screening, therapeutic colonoscopies performed, and treated patients for CRC during two periods.

**Results:**

In general, 1318 lower gastrointestinal endoscopic procedures were performed in the first group and 862 procedures in the second group, which was 34.6% less compared to the first group. The first group included 672 (51%) colonoscopies, 172 (13%) day surgeries and 474 (36%) CRC screening programmes. In group 2, 456 (34.6%) less patients underwent CRC diagnostics and treatment: 141 (21%) less colonoscopies, 93 (54%) less day surgeries, 222 (47%) less CRC screening programmes, and 26 (13%) less patients were hospitalized for surgical treatment (196 vs 170).

**Conclusion:**

Our study reveals worrying changes in the timely access to diagnostic procedures during the COVID-19 pandemic that possibly provoked rise in cases with the advanced stage CRC. However, despite numerical difference between groups existed, the difference between groups do not reach statistical significant level.

## Santrauka.

**Tikslas:**

mūsų darbo tikslas – įvertinti pirmosios COVID-19 pandemijos Lietuvoje poveikį storosios žarnos vėžio patikros programoms vykdyti, vėžio diagnostikai ir gydymui.

**Metodai:**

atliktas retrospektyvusis tyrimas, jo metu analizuoti Nacionalinio vėžio instituto duomenys. Šis tyrimas aprėpė į du periodus: nepandeminį periodą (nuo 2019 m. sausio 1 d. iki liepos 31 d.) ir pandeminį periodą (2020 m. sausio 1 d. iki liepos 31 d.). Analizuota pacientams, dalyvavusiems patikros programoje, atliktų gydomųjų kolonoskopijų ir gydytų pacientų skaičius per šiuos du laikotarpius.

**Rezultatai:**

pirmu periodu buvo atlikta 1 318 endoskopinių procedūrų, o antruoju – 862, arba 34,6 proc. mažiau. Pirmu periodu atliktos 672 (51 proc.) diagnostinės, 172 (13 proc.) dienos stacionaro ir 474 (36 proc.) patikros programos kolonoskopijos. Antruoju: 456 (34,6 proc.) mažiau diagnostinių ir gydomųjų procedūrų: 141 (21 proc.) mažiau diagnostinių, 93 (54 proc.) mažiau dienos stacionaro ir 222 (47 proc.) patikros programų kolonoskopijų. Be to, mažiau operuota pacientų (196 *vs* 170).

**Išvados:**

mūsų tyrimo duomenimis, pandemijos laikotarpiu sumažėjo diagnostinių procedūrų skaičius, o tai galimai lėmė didesnį lokaliai išplitusių vėžių kiekį. Nors ir buvo rezultatų skirtumų tarp periodų, tačiau statistinio patikimumo nenustatyta.

## Introduction

According to *Bray et al*, over 1.8 million new colorectal cancer (CRC) cases and 881,000 cancer deaths were estimated to occur in 2018 [1]. Overall, colorectal cancer ranked third in terms of the incidence and second in terms of mortality worldwide in 2018.

The COVID-19 pandemic significantly affected colorectal cancer (CRC) diagnostics and treatment [2, 3]. The first COVID-19 positive patient in Lithuania was confirmed on 28th February, 2020, while the lockdown regime in Lithuania was started on 16th March 2020. During the pandemic period, the nonurgent surgeries and medical procedures, such as colonoscopies and day surgeries, were delayed, and the access to the primary care facility and national CRC screening program was restricted. These changes in accessibility of health care service increased unmet needs of patients and possibly restricted timely diagnostics of early stage cancer. This could lead to CRC progression to incurable, metastatic cancer and affect both the quality of life and life expectancy of the patient.

CRC, which is preventable through screening, is the third most common cancer in Lithuania and the second most common cause of cancer deaths according to the Lithuanian Institute of Hygiene 2019 data [4, 5]. Recent studies suggest that the interruption of CRC screening program leads to a delayed diagnosis of CRC, while the restricted access to endoscopic procedures is evidently related to the growth of missed CRC cases [2, 3].

Our aim was to see the possible effect of the first COVID pandemic wave in Lithuania on colorectal cancer (CRC) preventive, diagnostic and treatment procedures.

## Methods

A retrospective analysis was performed using the database of the National Cancer Institute of Lithuania, the only specialized cancer centre in the country.

In this study, we retrospectively have assessed our hospital database. We included patients who underwent CRC screening program, colonoscopy, and day surgery or were hospitalized at the Department of Abdominal and General Surgery and Oncology for elective or emergency surgery. Patients for day surgery are admitted to the ward for not more than 24 hour surveillance. The indications for day surgery are the polyps of more than 1 cm or endoscopic submucosal dissection or resection. CRC screening program includes all faecal immunochemical test (FIT)-positive people of 50–74 years old. The colonoscopy contingent consisted of patients who underwent diagnostic colonoscopies alone or day surgery patients undergoing colonoscopies with polypectomies. We have divided patients into two groups: group 1 – patients treated during the nonpandemic period (2019 January 1 to 2019 July 31) and group 2 – the pandemic period (2020 January 1 to 2020 July 31). The impact of the restriction in diagnostic procedures, the changes in endoscopy numbers and stages of the CRC were evaluated.

Data were analyzed using the Statistical Package of Social Science (SPSS) program for Windows (Standard version 21; IBM). Absolute and relative numbers (percentages of the structure) for both periods are given. The two periods were compared by absolute and the consisting parts using the Student t-test. The results were considered significant when p ≤ 0.05.

## Results

**Figure 1.**
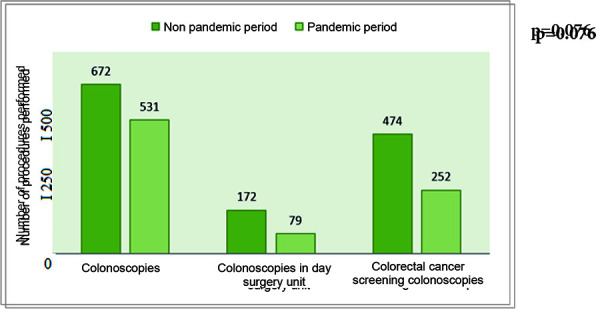
The numbers of the lower gastrointestinal endoscopic procedures for colorectal cancer in the nonpandemic and pandemic periods.

In general, 1318 lower gastrointestinal endoscopic procedures were performed in the first group and 862 procedures in the second group, which was 34.6% less compared to the first group. The first group included 672 (51%) colonoscopies, 172 (13%) day surgeries and 474 (36%) CRC screening programmes. The second group included 531 (62%) colonoscopies, 79 (9%) day surgeries and 252 (29%) CRC screening programmes (see Figure 1).

**Figure 2.**
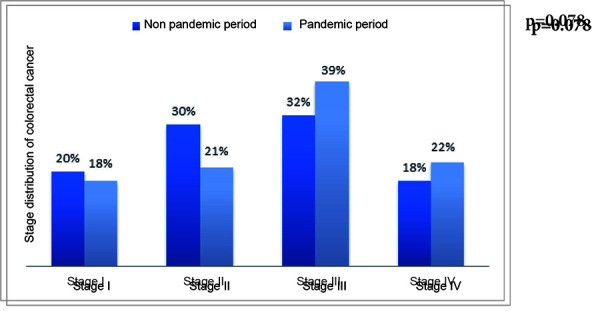
The stage distribution of the colorectal cancer in the nonpandemic and pandemic periods.

Overall, we had 196 patients in group 1 and 170 patients in group 2. With the stage I colorectal cancer, the first group included 40 (20%) patients compared to 30 (18%) patients in the second group, with stage II – 58 (30%) vs. 36 (21%), with stage III – 63 (32%) vs. 67 (39%) and with stage IV – 35 (18%) vs. 37 (22%) patients (see Figure 2).

In group 2, 456 (34.6%) less patients underwent CRC diagnostics and treatment: 141 (21%) less colonoscopies, 93 (54%) less day surgeries, 222 (47%) less CRC screening programmes, and 26 (13%) less patients were hospitalized for surgical treatment. Five (1%) patients were diagnosed with CRC in the screening program in group 1 compared to ten (4%) in group 2. During screening colonoscopies, polyps were found in 52 patients before pandemics compared to 30 during the pandemics.

**p=0.076**

**p=0.078**

## Discussion

COVID-19 disease affected health care services all around the world. A significant decrease in cancer diagnoses is seen comparing the pre-COVID era and the COVID one [6]. The decrease is determined by reduced number of consulted patients with nonspecific or nonacute potential symptoms of cancer, the consulting issues via telehealth, the delayed hospital referrals, the postponed diagnostic evaluations and halted national cancer screening programmes, and even the patients anxiety about acquiring the COVID-19 infection at hospitals. However, even though the diagnostic and treatment procedures in the nonpandemic period outnumbered the ones in the pandemic period by 1318 to 862, there was 4 times more CRC diagnoses confirmed during COVID-19 outbreak in our hospital – 5 (1%) to 10 (4%) CRC diagnosed patients.

In Lithuania, the CRC screening program was stopped for one month during pandemic. At National Cancer Institute there was stoppage of two weeks. Moreover, there were transport restrictions on traveling between the municipalities. Decreased number of buses and trains between the cities was noticed. Moreover, the number of consultations of the specialists and general practitioners were significantly decreased and the breaks between the visits were increased. This led to significant decrease in numbers of referrals to our institute. In addition, people with symptoms were afraid to go to hospitals. This also delayed the diagnosis and treatment of CRC for few months. It was reported that in 2020 we could expect around 4,000 new CRC cases for each month of the epidemic in Italy [2]. The similar scenario can be expected in Lithuania. The endoscopic cancer diagnostics was also remarkably reduced during the COVID-19 period causing concerning cancer detection decrease [3]. Unsurprisingly, our study shows that the rate of patients with III-IV CRC stages increased by 12% during the pandemic period. Cancer stage is one of the main factors influencing cancer survival.* Lee at al.* recommend that the interval from diagnosis to treatment for all CRC patients, regardless of disease stage, should not exceed 30 days [7]. Authors analyzed three groups by diagnosis to treatment interval: ≤30 days (90.5% patients), 31–150 days (6.4% patients) and ≥151 days (3.15% patients), and the risk of death was consistent across all cancer stages and increased for the second (hazard ratio 1.51; 95% confidence interval 1.43–1.59) and the third (1.64; 1.54–1.76) groups compared to the first one. In our study, the number of the hospitalized patients with CRC decreased by 13% during the pandemic, meaning that not all the patients were referred for the treatment. The delay of primary elective curative surgical treatment of CRC affects patient’s overall survival [8, 9]. *Grass et al.* study compared overall survival in the short treatment delay group (<16 days, 31,171 patients) and long delay group (≥37 days, 29,617 patients). Overall survival in both groups, respectively, was 75.4 vs. 71.9% at 5 years and 56.6 vs. 49.7% at 10 years (both p<0.0001). The delay of three and more months from time of diagnosis significantly increases the adjusted hazard ratio of mortality (1.4 times) [8]. For this reason, *Grass et al.* encourages the surgical treatment within 40 days following the initial diagnosis. To prevent the cancer deaths, diagnostics and surgeries must be maintained at usual efficiency and necessary attention intended to postpone cases. During pandemic, *Nodora et al.* recommend performing faecal immunochemical test at home and deliver it to hospital by mail [10].

To ensure effective communication with patients, telehealth-based care should be improved. The general practitioner would need to monitor FIT-positive symptomatic patients and prioritize out of delay for colonoscopy [11]. Colonoscopy is a very important tool in CRC screening and treatment; therefore it must be continued even during the COVID-19 period with precautions. The British Society of Gastroenterology Endoscopy Committee and Quality Improvement Programme guidelines recommend patients undergoing telephone call on symptoms using systematic questionnaires before endoscopy during COVID-19 period [12]. Moreover, all outpatients should undergo genetic (PCR) testing for COVID-19 virus 1–3 days prior to endoscopy and PPE should be determined by patient risk stratification. In resuming endoscopy procedures, patients; and staff protection should be ensured.

Our study obviously has some limitations. First, this is a single centre rather small sample size, retrospective study. Moreover, the long-term effect of all the delays should be analyzed in future. In addition, possible changes in treatment modalities should be assessed.

To conclude, our study reveals worrying changes in the timely access to diagnostic procedures during the COVID-19 pandemic that possibly provoked rise in cases with the advanced stage CRC. However, despite numerical difference between groups existed, the difference between groups do not reach statistical significant level. Long-term results should be awaited. Specific pathways, scenarios and models prioritizing certain patients and diseases must be introduced in future.

## Declarations

**Funding –** none

**Conflicts of interest/Competing interests** – none

**Ethics approval** – approved by institutional review board.

**Authors’ contributions** – AD and SJ conceived the idea of the study. AD, SJ, IK, ES, DJ, SM designed the article methodology and gathered the data. DJ, SM performed the data analysis and drafted the article. AD, IK and EK conducted screening of articles and data extraction. All authors critically reviewed subsequent drafts.
